# ECOD: identification of distant homology among multidomain and transmembrane domain proteins

**DOI:** 10.1186/s12860-019-0204-5

**Published:** 2019-06-21

**Authors:** R. Dustin Schaeffer, Lisa Kinch, Kirill E. Medvedev, Jimin Pei, Hua Cheng, Nick Grishin

**Affiliations:** 1 Department of Biophysics, University of Texas Southwestern Medical Center, Dallas, TX 75390-9050 USA; 2 Howard Hughes Medical Institute, University of Texas Southwestern Medical Center, Dallas, TX 75390-9050 USA

## Abstract

The manual classification of protein domains is approaching its 20th anniversary. ECOD is our mixed manual-automatic domain classification. Over time, the types of proteins which require manual curation has changed. Depositions with complex multidomain and multichain arrangements are commonplace. Transmembrane domains are regularly classified. Repeatedly, domains which are initially believed to be novel are found to have homologous links to existing classified domains. Here we present a brief summary of recent manual curation efforts in ECOD generally combined with specific case studies of transmembrane and multidomain proteins wherein manual curation was useful for discovering new homologous relationships. We present a new taxonomy for the classification of ABC transporter transmembrane domains. We examine alternate topologies of the leucine-specific (LS) domain of Leucine tRNA-synthetase. Finally, we elaborate on a distant homologous links between two helical dimerization domains.

## Background

The classification of protein structures deposited in the PDB increasingly involves complexes, transmembrane proteins, and multidomain proteins with non-globular internal repeats [1]. This trend is partly due to the improvement of structural determination techniques using cryo-electron microscopy and of transmembrane proteins by X-ray crystallography [2, 3]. Through covariation-based structure prediction, there are likely few remaining soluble, globular, protein structures that are not predictable computationally [4, 5]. Consequently, those structures which are targeted for structural determination and which cannot be easily classified tend to be transmembrane and/or or large multidomain structures participating in a protein complex. Although the number of such unpredictable proteins is small, they can be expected to disproportionately be revealed as targets for manual curators in any knowledge-based structural protein classification.

**Fig. 1:**
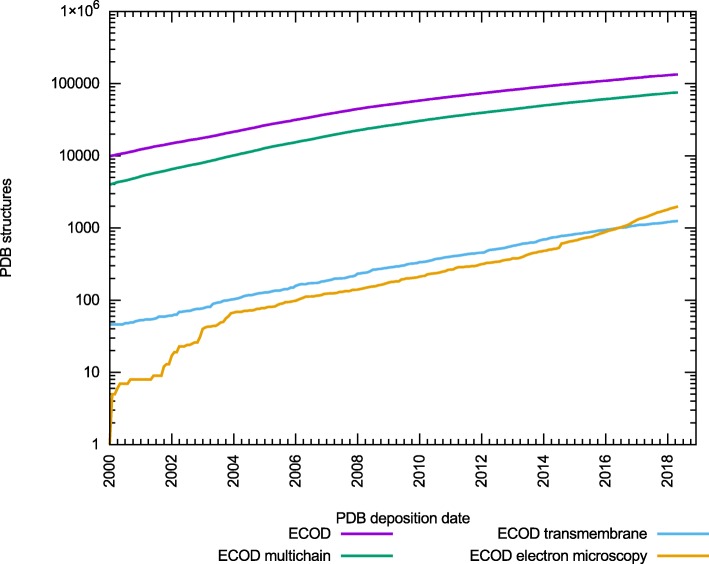
Increase of complex structures classified by ECOD. The distribution of protein structures classified by ECOD (red) that are either multi-chain (green), contain at least one transmembrane regions (blue), or are determined by electron microscopy techniques(cyan). The rate of increase of each of these types of structure is similar

We have previously described ECOD (Evolutionary Classification Of protein Domains), our comprehensive classification of protein structures and their domains [6]. Briefly, the principal variation of ECOD from other structural classifications is its reliance on evolutionary relationships, rather than topology, as its basic organizing theory. Like other structural classifications (SCOP, SCOPe, and CATH), ECOD relies on a mixed manual/automatic methodology to incorporate new structures into the classification as they are determined [7–10]. This mixed approach allows us to access the consistency, speed, and reproducibility of an automated approach while also incorporating the ingenuity and intuition of manual curation. The incorporation of both approaches also acts as a check against systematic errors, automated methods can help detect manual inconsistencies, and manual curation can assist in detection of false positive automated cases. Increasing examples of large protein complexes containing multichain and multidomain arragements have altered the types of classification problems faced by manual curators (Fig. 1).

Here we present five case studies of protein classification where automated approaches failed, but manual curation resolved the classification. These cases were chosen from a period of over 2 years of manual curation of ECOD and reflect the current state of the manual curation of our classification. Two cases illustrate the complications of the classification of transmembrane domains. The ABC transporter transmembrane domains are ubiquitous proteins with complex evolutionary origins. We present a case where a previous homologous link was judged insufficient for classification, as well as a novel structure which joined previously unlinked ECOD homologous groups. The final two cases reflect the difficulty of classifying homologous multi-domain proteins with variant topology within their conserved domain architecture. We show how we identified the Leucine-specific domain (LS) of leucine tRNA-synthetase (LeuRS) is topologically distinct between orthologs and likely a detiorated zinc ribbon domain descended from rubredoxin-like domains. Additionally, we judge that the dimerization domain of PAN3 is homologous to the human Caprin-1 dimerization domain based on topological and functional similarity.

## Results and discussion

### Manual curation of protein classification

The mission of ECOD is the comprehensive classification of the proteins with known three-dimensional structures. Our target is the Protein Data Bank (PDB), the current global resource for the deposition of protein structures. Since the release of ECOD in 2014, we have pursued a policy of weekly updates, using a combined automated pipeline and manual curation workflow. Although our full automatic methodology is described elsewhere [6, 11, 12], we briefly elaborate on the conditions for successful classification. A combination of sequence-based aligners is used to determine putative boundaries by alignments against an ECOD-derived reference database. Where a series of domain alignments can be generated against a query that a) cover the query sequence completely (or nearly so) and b) do not overlap with each other, the putative domain assignments for the query chain are accepted. Where the automated pipeline cannot generate a complete putative assignment, it is because a) consistent domain boundaries for known domains cannot be generated, b) some region of the query does not have known homology to our reference, c) and/or because some regions of the query are practically or technically unassignable (they are made up of unknown residues, they are fragments of known domains, they are synthetic constructs with no evolutionary history, etc). Where one or more of these problems occur in a query, the protein in question is flagged for manual curation.

We analyzed 2 years of weekly and semi-weekly updates between 2016 and 2018 to examine the outcome of our manual curation decisions. Over this period, representative proteins chains were selected from a set of proteins for which the automated domain partition and assignment pipeline failed to generate complete domain assignments. This representative set (70% sequence identity) conained 2298 protein chains contained within 1656 PDB depositions over 120 updates. The principal curation judgement is whether a protein chain contains non-domain regions. 1558 (67%) protein chains were wholly or in part were judged unclassifiable as domains. 435(18%) were entirely classified into an ECOD special architecture (peptide, fragment, synthetic peptide, etc.), whereas only 1123(48%) were classified in part as special architectures. Of these representative chains, 1351(58%) contained at least one region that was partitioned and assigned into a domain by manual curators. Additionally, 732(31%) protein chains classified by manual curators into domains consisted of only a single domain (and potentially some unclassifiable region), whereas 619 (26%) protein chains were determined to be multi-domain proteins. 119 (5%) proteins requiring curation contained at least one transmembrane segment (as determined by PHOBIUS. 1335 (58%) were from structures determined by X-ray crystallography, whereas 802 (35%) were determined by electron microscopy. These decisions to split and assign representative chains are the primary source of the curation examples discussed below.

### Type II CAAX protease homolog and γ-secretase subunit APH-1

Eukaryotic type II CAAX prenyl endopeptidases, also named Ras and a-factor converting enzymes (RCE1), are integral membrane proteins that catalyze the removal of the “AAX” tripeptide from the CAAX motif (C:cysteine, A:aliphatic residue, X:C-terminal residue) after the prenyl attachment to the cysteine residue [13]. Distantly related members of this family, subsequently named CPBP [14], have been found in numerous bacterial and archaeal organisms. These proteins possess the EEXXXR motif in one of the transmembrane (TM) segments and two conserved histidines residing in another two distinct TM segments. While conservation of the glutamates and histidines are reminiscent of active site composition of zinc-binding metalloproteases [13], the structure of an archaeal homolog of type II CAAX prenyl proteases revealed no metal binding sites [15]. These intramembrane proteases are thus more likely to be glutamate proteases, consistent with the observed lack of effect of metalloprotease inhibitors on their catalysis. This Rce-1 structure (PDB:4cad) was subsequently classified in ECOD as a novel X-group, a structure possessing no homology to any other known structural domain.

**Fig. 2:**
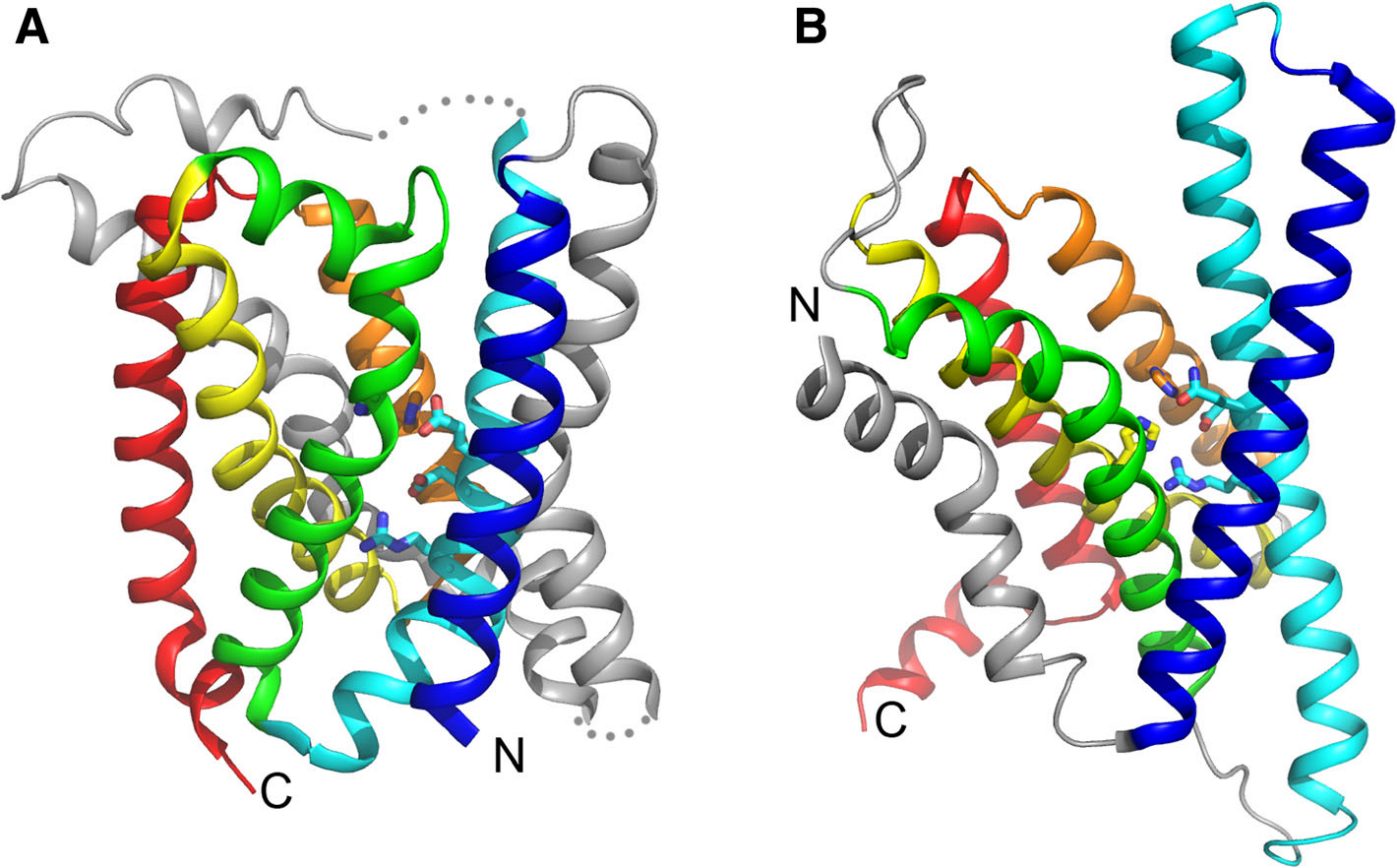
Homology between APH-1 and type II CAAX protease homolog. The structures of type II CAAX protease homolog (pdb: 4CAD, chain C) and γ-secretase subunit APH-1 (pdb: 5A63, chain C) are shown in panels **a** and **b**, respectively. Six core TM segments shared between them are colored blue, cyan, green, yellow, orange, and red from N- to C-terminus. Sidechains of conserved polar residues inside membrane are shown, including EExxxR motif in core TM segment 2 and two histidines in core TM segments 3 and 5 for type II CAAX protease homolog and QExxR motif in TM segment 2 and two histidines in core TM segments 4 and 5 for APH-1

Remote homologs to CPBP family proteases were identified by sensitive profile-profile comparisons, including the eukaryotic γ-secretase subunit APH-1 and the bacterial PrsW proteases [14]. These proteins contain sequence motifs in four core transmembrane segments, such as EExxR and QExxR, that can be aligned to the EEXXXR motif in CPBP family proteases. They also share conserved histidines and some positions with small conserved residues. The cryo-EM studies of the γ-secretase complex [16, 17] revealed the three-dimensional structure of APH-1, which exhibits an overall fold similarity to the archael CPBP member, as well as conserved polar groups inside the transmembrane segments. These two structures share six core transmembrane segments arranged in the same topology (Fig. 2). Consecutive TM segment triplets are right-handed for TM segments 1,2,3 and 2,3,4, and left-handed for TM-segments 3,4,5 and 4,5,6. APH-1 has an additional N-terminal TM segment (colored grey in Fig. 2a), whereas archaeal CAAX protease homolog possesses two extra TM segments between TM1 and TM2 (colored grey in Fig. 2b). Conserved polar residues are observed in TM2, TM3, TM5 for archaeal CAAX protease homolog and TM2, TM4, TM5 from APH-1. Based on this homology, we classified APH-1 as homologous to the type II CAAX protease homolog and placed them in the “RCE-1-like” H-group in ECOD.

### The unified ABC transporter transmembrane domain X-group

ABC (ATP-binding cassette) transporters are a diverse group of proteins or protein complexes that couple the energy generated from ATP hydrolysis to transport/translocate various cargo molecules [18]. ABC transporters share the homologous nucleotide-binding domains (NBDs) for ATP binding and hydrolysis. On the other hand, the transmembrane domains (TMDs) of ABC transporters are structurally diverse and can possibly have different evolutionary origins, e.g., type I ABC importer TMDs (Pfam family: BPD_transp_1) and type II ABC transporter TMDs (such as Pfam families BPD_transpd_2 and FecCD in Pfam clan Membrane_trans) [19, 20].

**Fig. 3:**
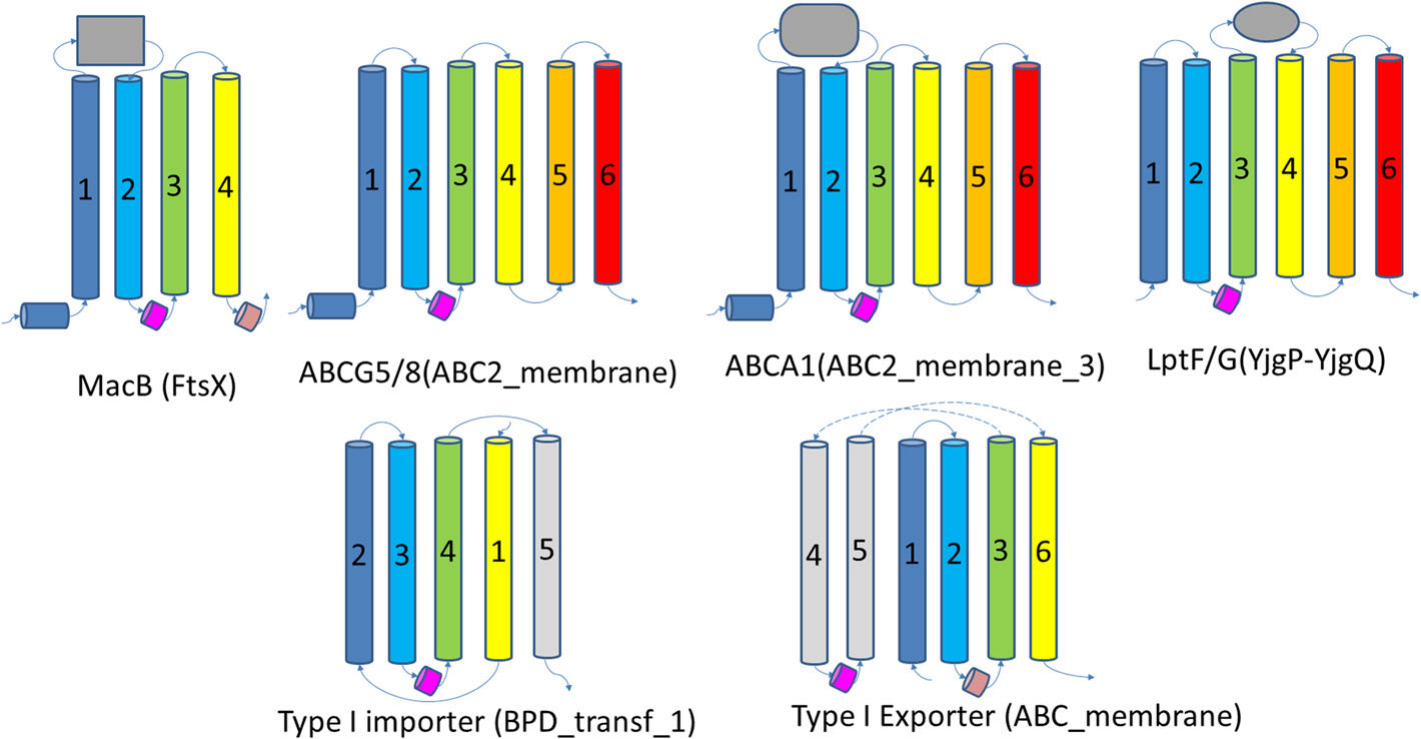
Homologous transmembrane topologies among type II ABC transporter transmembrane domains. Cartoon diagrams for ABC transporter TMDs in the same X group. The four shared TM segments with the same fold among them are colored blue, cyan, green, and yellow from N- to C-terminus. Two additional C-terminal TM segments are colored orange and red in ABCG, ABCA, and LptF/G structures. Other TM segments are colored gray. The TM segments are sequentially numbered from N- to C-terminus for each structure. Canonical coupling helix shared among them are colored magenta, and the additional coupling helix is colored pink for MacB and type I exporter. Substrate-binding domains in between TM segments are shown as gray objects for MacB, ABCA1, and LptF/G. Top panel contains the H group type II exporters. Pfam family names are shown in parentheses. These diagrams are based on these structures: MacB - 5ws4; ABCG5/8 - 5do7; ABCA1 - 5xjy; LptF/G - 5x5y, type I importer - 3dhw; and type I exporter - 5mkk

Three non-canonical ABC transporter TMD domains: FtsX, YjgP_YjgQ, and DUF1430 – have been classified in the Pfam clan BPD_transp_1 along with the type I importer TMD (Pfam family BPD_transp_1). The structures of members of the FtsX [21–24] and YjgP_YjgQ [25, 26] Pfam families have been solved recently, as well as members of type II exporters such as ABCG [27–29] and ABCA [30], proteins involved in the export of lipid molecules in eukaryotes. Interestingly, these structures exhibit high similarities, suggesting that they might be evolutionarily related. The core of these structures consists of four core TM segments, as observed in several structures (e.g., PDBs5ws4 and 5xu1) of the MacB family of ABC transporters with the FtsX domain. A coupling helix (Fig. 3; magenta) from the MacB TMD resides between the second and third core TM segments. The handedness of consecutive TM segment triplets are both right handed for the first three TM segments and the last three TM segments. Such a core structure (blue, cyan, green, yellow segments in Fig. 3), together with the location of the coupling helix, is also present in structures of the YjgP_YjgQ (e.g., PDBs 5x5y and 5 l75), ABCG (e.g., PDBs 5do7 and 6ffc), and ABCA (PDB: 5xjy)TMDs, which each have two additional C-terminal TM segments (Fig. 3, orange and red). MacB additionally possesses a second coupling helix at the C-terminal end of the fourth core TM segment (Fig. 3, pink), which is absent from YjgP_YjgQ, ABCG, and ABCA TMDs. We consider these families of ABC TMDs to be homologous and classified them as a single homologous group (H: ABC type II exporter TMDs?).

The six TM segments of YjgP_YjgQ, ABCG, and ABCA TMDs adopt the same topology as the core domains of APH-1, as suggested by structure similarity searches. For example, the DaliLite search using the YjgP_YjgQ TMD as a query (PDB:5x5y, chain F) found the APH-1 structure (PDB:5fn2,chain C) with a Z-score of 8.9, the best score other than other YjgP_YjgQ domains. However, these ABC transporter TMDs do not have the conserved polar residues observed in the active site of APH-1 and type II CAAX proteases. It is unclear that these ABC transporter TMDs and APH-1/type II CAAX are homologs. Given their functional dissimilarity, we chose not to classify them in the same X-group.

The type I ABC importer TMD (Pfam: BPD_transpd_1) is structurally similar to MacB (Pfam:FtsX) if its first TM segment is considered to structurally occupy the position of the last TM segment of the core of MacB/YjgP_YjgQ/ABCG/ABCA (H: type II exporter) with a coupling helix (Fig. 3, pink) in between the second and third TM segments. Such a circular permutation event was predicted in our previous study of ABC transporters [31] and was confirmed with the recently solved MacB structures [21–24]. Type I ABC exporter TMD also exhibits structural similarity to type II ABC exporter domains. For example, the TMD of MacB (PDB:5ws4) identified type I exporter (PDB:5mkk) as one of its top hits (Dali Z-score, 7.7). The fourth and fifth TM segments of type I exporter TMD (Fig. 3, gray) are swapped between two TMD units and contain the canonical coupling helix (Fig. 3, magenta), while TM segments 1,2,3, and 6 of type I exporter can be structurally aligned to the four TM segments of MacB. We classify the type I importer, type I exporter, and type II exporter TMDs as three H-groups in the same X-group (X: Type I ABC importer and type I/II ABC exporter TMDs), as they might be remote homologs based on their structural similarity and common function in ABC transporters. This X-group also contains an H-group of multidrug exporters discussed below.

### MatE: predicted structures of an efflux toxin aid in classification

The multidrug and toxic compound extrusion (MatE) family includes integral membrane proteins that couple electrochemical gradients to export of metabolites across the cell membrane leading to multidrug resistance (MDR) in bacteria and animals and disease resistance in plants. MatE transporters belong to a larger multidrug/oligosaccharidyl-lipid/polysaccharide (MOP) exporter superfamily [32]. The MOP family also includes two major clusters of prokaryotic polysaccharide transporters (lipopolysaccharide O-antigen exporters and exopolysaccharide exporters) as well as eukaryotic oligosaccharidyl-lipid flippases.

**Fig. 4:**
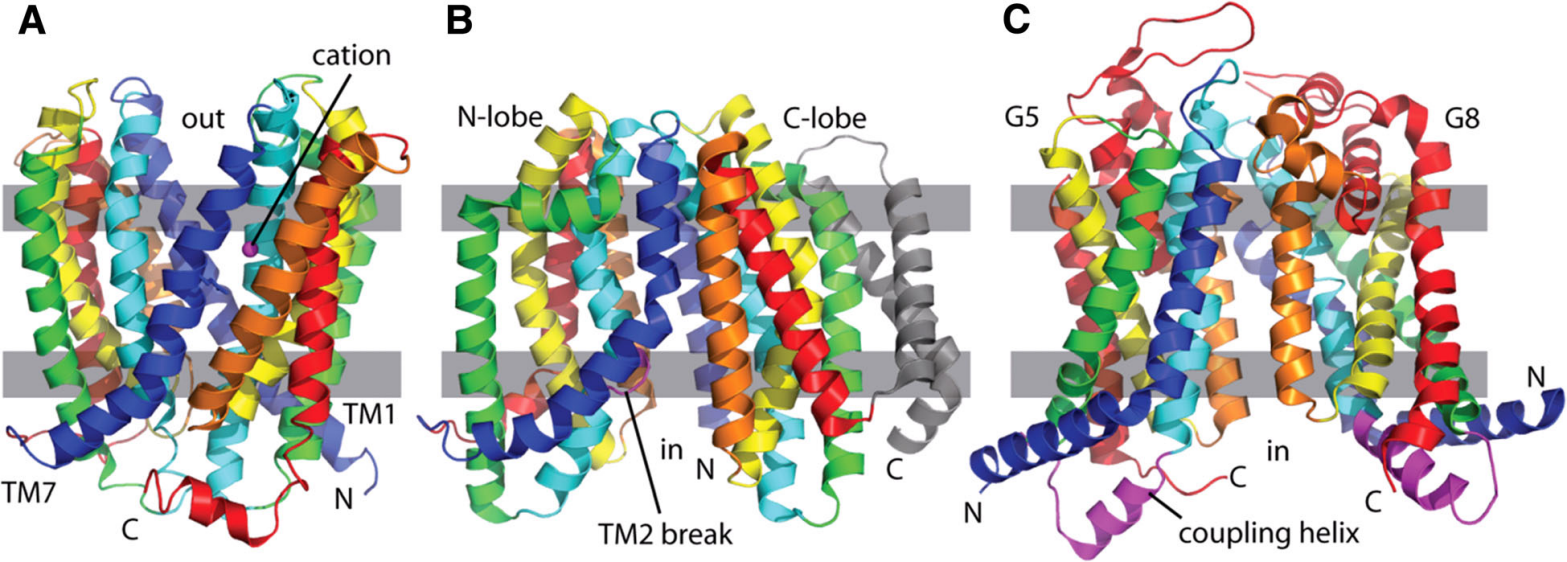
Homology between MatE transporter and ABC2 transmembrane domains. Transporters are depicted in cartoon, with the cytoplasm on the bottom and transmembrane region marked by grey boxes. **a** MatE-like MDR efflux pump (PDB: 3mku) exhibits an internal duplication of two transmembrane bundles (TMs 1–6 and TMs 7–12), with each colored in rainbow from blue to red. A bound monovalent cation (magenta sphere) highlights an outward-facing binding site for molecules of the electrochemical gradient. **b** The MOP superfamily member MurJ flippase (PDB: 5 t77) adopts a similar overall duplicated topology with a C-terminal two TMH extension to the C-lobe and an inward-facing topology. A sequence motif (magenta) marks a broken TM2 that might contribute to transport. **c** The ABC2 transmembrane domains from the ABCG5/8 heterodimer (PDB: 5do7) retains a similar overall core topology, except with swapped N-terminal helices. The functional coupling helix (magenta) follows TM2

A crystal structure of the MatE-like *Vibrio cholerae* MDR efflux pump NorM (PDB: 3mku) adopts a duplication of six TM helices (TMs 1–6 and 7–12) arranged as two domains (Fig. 4a) [33]. The duplicated NorM domains are open to the extracellular space, and the structure is bound to a monovalent cation responsible for transport function [33]. Subsequent structures of substrate-bound MatE transporters revealed that the outward-facing cavity as the multidrug binding site [34]. MatE transporters are thought to couple H^+^ or Na^+^ gradients with drug extrusion in an alternating access cycle of outward-facing and inward-facing conformations. The sequence related MurJ lipid flippase structure (PDB: 5 t77) adopts a similar core 12 TM topology as MatE (Fig. 4b), with a C-terminal extension of two TM helices in the C-terminal domain. In contrast to available MatE structures, the MurJ N-terminal domain (TMs 1–6) and C-terminal domain (TMs 7–12) are arranged in an inward-facing conformation. This alternate conformation is marked by an asymmetry between the two domains with TM helix 1 extending out from the TMD core rather than interacting with the C-terminal domain and TM helix 2 being broken at a conserved sequence motif [35].

The overall MatE topology resembles that of type II ABC exporter TMDs (ABC2) exemplified by the inward-facing ABCG5/ABCG8 heterodimer structure (Fig. 4c). The relationship between these two distantly related structures can be detected by residue covariation [4]. For example, a structure model of the ABC2 heme exporter protein B dimer (CcmB) built using residue covariation restraints [36] is similar to both the MatE and Type II ABC exporter transmembrane domain structures (Table 1). Notably, using representative structures from the MatE or Type II ABC exporter folds (or the CcmB model) as queries, all of the top-scoring structures in the database belong to these two topologies. A comparison of the two previously distinct ECOD X-groups that are related to the CcmB structure model reveals thateach ABCG subunit represents one domain of the MatE transporters, except with swapped N-terminal helices. The functional helix that couples the ABCG TM domain to the intracellular NBD resides at the C-terminus of TM helix 2 [27]. Interestingly, the ABCG coupling helix resides at the same position as the helix following the sequence-conserved break in MurJ TM helix 2. This unusual structural feature, together with the presumed adoption of alternating inward-facing and outward-facing conformations for transport, supports the notion that these two distantly related families are homologs. Potentially, the type II ABC exporters substituted electrochemical gradient-dependent export with transport driven by ATPase activity of the intracellular NBD. As such, we classify the MatE exporter H-group inside of the ABC transporter TMD X-group.

**Table 1:** Top DaliLite results using CcmB as a query against the PDB25 dataset

Query	Hit	Z-score	Protein	Old X-group
CcmB	4mlb-B	8.8	PF0708	MatE transporter
CcmB	2yvx-A	8.2	MG2+ TRANSPORTER MGTE	MatE transporter
CcmB	5xjy-A	8	ATP-BINDING CASSETTE SUBFAMILY A MEMBER 1	Type II ABC exporter
CcmB	6an7-C	8	ABC TRANSPORTER	Type II ABC exporter
CcmB	5yck-A	7.2	MULTI DRUG EFFLUX TRANSPORTER	MatE transporter
CcmB	5do7-A	7.2	ATP-BINDING CASSETTE SUBFAMILY G MEMBER 5	Type II ABC exporter
5x5yF	5x5y-F	46.6	ABC transporter LptB2FG	Type II ABC exporter
5x5yF	5l75-G	23.3	LIPOPOLYSACCHARIDE ABC TRANSPORTER	Type II ABC exporter
5x5yF	5l75-F	23	LIPOPOLYSACCHARIDE ABC TRANSPORTER	Type II ABC exporter
5x5yF	5x5y-G	19.6	ABC transporter LptB2FG	Type II ABC exporter
5x5yF	5a63-C	9.5	NICASTRIN	Type II ABC exporter
5x5yF	4mlb-B	8.1	PF0708	MatE transporter
5x5yF	5xjy-A	8.1	ATP-BINDING CASSETTE SUBFAMILY A MEMBER 1	Type II ABC exporter
5x5yF	5yck-A	7.7	MULTI DRUG EFFLUX TRANSPORTER	MatE transporter
5x5yF	3mkt-A	7.6	MULTI ANTIMICROBIAL EXTRUSION PROTEIN	MatE transporter
5x5yF	5do7-A	7.5	ATP-BINDING CASSETTE SUBFAMILY G MEMBER 5	Type II ABC exporter
4mlbB	4mlb-B	70.4	PF0708	MatE transporter
4mlbB	5yck-A	41.7	MULTI DRUG EFFLUX TRANSPORTER	MatE transporter
4mlbB	3mkt-A	36.2	MULTI ANTIMICROBIAL EXTRUSION PROTEIN	MatE transporter
4mlbB	6cc4-A	14.2	SOLUBLE CYTOCHROME B562, LIPID II FLIPPASE MURJ C	MatE transporter
4mlbB	5l75-F	8	LIPOPOLYSACCHARIDE ABC TRANSPORTER	Type II ABC exporter
4mlbB	5x5y-F	7.3	ABC transporter LptB2FG	Type II ABC exporter
4mlbB	5x5y-G	7.1	ABC transporter LptB2FG	Type II ABC exporter

### Leucine-specific domain in leucyl-tRNA synthetase

**Fig. 5:**
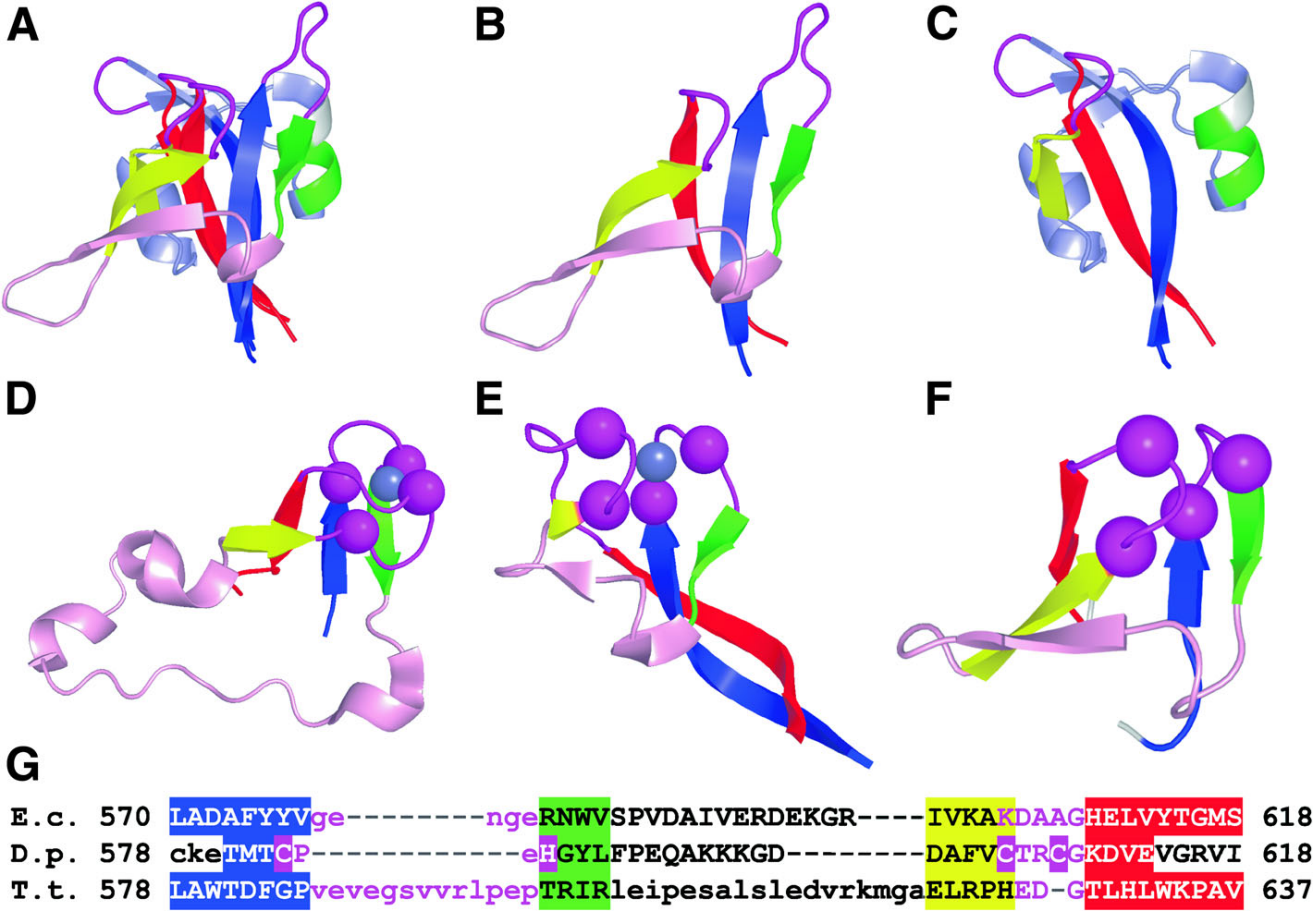
Structural and functional similarity between leucine-specific domain of LeuRS and rubredoxin-related zinc ribbons. **a** – structural alignment of leucine-specific domains of *E.coli* LeuRS (PDB: 4AQ7) and *T.thermophilus* LeuRS (PDB: 1H3N). Aligned regions colored by respective colors, unaligned regions colored by pale pink (*E.coli* LeuRS) and pale blue (*T.thermophilus* LeuRS). Structures of LeuRS leucine-specific domains of *E.coli* (**b**), *T.thermophilus* (**c**). **d**, **e** - Structures of *T.thermophilus* LeuRS zinc binding domains. Cα atoms of residues which take part in Zn binding are shown as spheres and colored in magenta. **f** - Rosetta model of leucine-specific domains of *D.phosphitoxidans* LeuRS. Residues which supposed to bind zinc are shown as spheres and colored in magenta. **g** – sequences alignment LeuRS leucine-specific domains, where *E.c.* - *E.coli*, *D.p.* - *D.phosphitoxidans*, *T.t.* - *T.thermophilus*. Residues which supposed to bind zinc are shown by magenta rectangles. Colored regions correspond to structurally aligned regions

Leucyl-tRNA synthetase (LeuRS) is a multi-domain class Ia aminoacyl-tRNA synthetase whose main function is to synthesize Leu-tRNA^Leu^ for use in protein synthesis. LeuRS consists of a main enzymatic component (composed of a Rossmann-fold catalytic domain and a class Ia-anticodon binding domain) and four additional flexibly linked domains, one of which is the leucine-specific domain [37]. The leucine-specific (LS) domain is located between the catalytic and anticodon-binding domains, N-terminal to a conserved KMSKS signature motif that binds tRNA [38]. Among known three-dimensional protein structures, the LS domain adopts two distinct topologies. The first (LS1) topology is represented by the LS domain of *E. coli* LeuRS (PDB: 4AQ7). This domain forms a two-layer β-sandwich with a β-sheet (antiparallel strands 1, 2, and 5) and a β-hairpin separated by a small α-helix between strands 2 and 3 (Fig. 5a, b, c). The second topology (LS2) can be observed in a LeuRS ortholog from *T. thermophilus* (PDB:1H3N). In this case, β-strands 1 and 5 are twisted around each other, the alpha-helix between strands 2 and 3 is significantly longer and additional alpha-helix is between strands 3 and 4. Despite these differences, structures of the two topologies can be superimposed (Fig. 5a), with both the β-1,2,sheet and the β-4,5 loop aligning well. The largest difference between these two topologies is following the first α-helix, with strand 3 and 4 from LS2 exiting in a different direction relative to the β-1,2,5 sheet (Fig. 5a).

The main function of LS is still unclear due to its variability (and its absence, in some cases) [39], but this domain is near the tRNA in different conformations of LeuRS structures. The unique β-4,5 hairpin of LS1 directly interacts with tRNA at the base of the D-loop stem in the editing conformation of *E. coli* LeuRS [37]. Similarly, the unique α-helix located between β-4 and β-5 of LS2 domain is located near the base of the tRNA D-loop (within 6 Å) in the exit complex of *T. thermophilus* LeuRS [39]. We note that LeuRS also contains two rubredoxin-related zinc ribbon domains that bind zinc using two loops between β-hairpins, and their common topological elements resemble those of rubredoxin-related zinc ribbons. Although LS domains lack zinc-binding residues, we hypothesize that LS is derived from a rubredoxin-related zinc ribbon. Using PSI-BLAST [40], we identified bacterial LeuRS that contain cysteines in their corresponding LS domain sequence. Using Rosetta [41], we performed de novo structure prediction for a LS domain of one such protein (*Desulfotignum phosphitoxidans*, NCBI ID: WP_006965092.1). The Rosetta model we chose as our representative showed that three cysteines and one histidine face inwards towards a putative zinc-binding site from the corresponding zinc-binding loops (Fig. 5f). Thus, our predicted Rosetta model supported our hypothesis that the LS domains are linked to zinc ribbons. Moreover, these data suggest that the LS domain is derived from a zinc ribbon domain that has lost its zinc-coordinating residues during evolution. Based on this analysis, we divided the LS domains classified within ECOD into two topology groups (T:LS1 and T:LS2), and moved the LS H-group into the rubredoxin –related X-group.

### Dimerization domains in PAN3 and Caprin-1

The poly(A)-specific nuclease (PAN) complex catalyzes mRNA deadenylation, a process in RNA degradation wherein AMP is released from the 3′ poly(A) tail of the mRNA substrate [42]. PAN contains two subunits, the exonuclease PAN2 and the adapter PAN3 [43]. Here we focus on the predominantly α-helical PAN3 C-terminal region (PDB: 4CYI,D) [44]. This region both homodimerizes and associates with PAN2 to assemble the functional PAN complex [43, 45]. We refer to this region as the PAN3 dimerization domain or PAN3 DD hereafter.

Caprin (cytoplasmic activation/proliferation-associated protein) is a small family of proteins that appear quite isolated in protein sequence space (ref? Why?). Caprin-1 participates in various cellular functions through its interactions with RNA and other proteins [46]. Recently, the structure of an alpha-helical dimerization domain (PDB: 4wbe) in Caprin-1 was reported [46]. Also determined was the highly similar structure of another family member’s dimerization domain (PDB: 5j97). In addition to homodimerization, Caprin-1 DD interacts with fragile X mental retardation protein (FMRP) [46, 47].

**Fig. 6:**
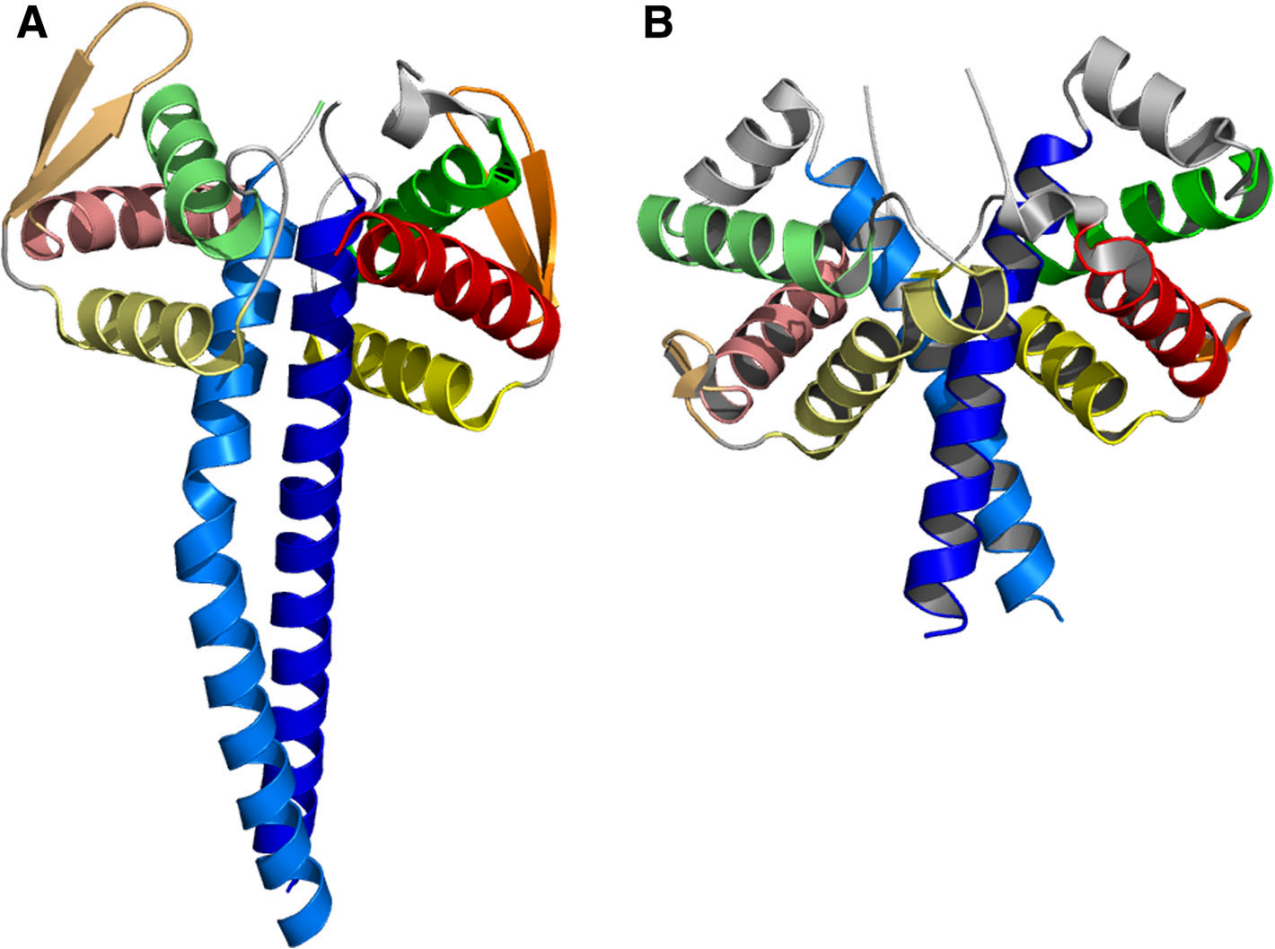
Structural similarity between PAN3 and Caprin-1 dimerization domains. PAN3 dimerization domain (PAN3 DD) and Caprin-1 dimerization domain (Caprin-1 DD) structures. **a** PAN3 DD dimer (PDB 4cyi, chains C and D, residues 498–631). **b** Caprin-1 DD dimer (PDB 4wbe, chains B and C, residues 133–251). Structurally corresponding SSEs in PAN3 DD and Caprin-1 DD are in the same color. Loops and noncorresponding SSEs are in gray. Each monomer is colored in rainbow from N-terminus (blue) to C-terminus (red). The two monomers in a dimer are colored in the same way except one is darker and the other is lighter

Although the authors considered Caprin-1 DD to have no structural similarity to existing structures, we found that it exhibits significant structural similarity to the previously published PAN3 DD structures. For example, a Dali search using Caprin-1 DD as a query returns multiple good hits to ECOD domains of PAN3 DD (Z-score ~ 8, RMSD ~ 3.7 Å). Indeed, PAN3 DD and Caprin-1 DD share a common topology composed of four major α-helices arranged in similar positions and angles (Fig. 6). A single β-hairpin is found preceding the final major helix in both structures: longer in PAN3 DD, much shorter in Caprin-1 DD. In addition to structural similarity, in both structures homodimerize via coiled-coils formed by their long, initial helices, although the two coils have opposite handedness (Fig. 6). Functionally, through both dimerization and interaction with other proteins, both PAN3 DD and Caprin-1 DD appear to serve as platforms for assembly of biological complexes [45, 46]. Based on these similarities, we hypothesize that PAN3 DD and Caprin-1 DD are evolutionarily related and represent a family of small, helical domains which served as scaffolds in other complexes. In ECOD, we place both domains in a single H-group (H: Dimerization domain in caprin-1 and PAN3).

## Conclusions

The classification of protein domains has shifted from a model where the identification of novel topologies and functions associated with those topologies is a principal goal, to a model where the most attention must be focused on large protein complexes, including those with transmembrane regions, and the boundaries and interactions of domains within these complexes. ECOD, our protein domain classification, shares an intellectual ancestry with other structural domain classifications. Our mixed manual/automatic classification pipeline, ensures that protein that cannot be classified automatically are curated manually. As part of this process, we here presented five cases initially identified by manual curators and their subsequent analysis to discover homologs. These cases were selected as representative of the types of difficult cases currently encountered by manual curators. We confirmed a previous prediction (based on sequence alone) regarding the homology between Rce-1 and APH-1. Based on novel structures, we identified novel homologs of the ABC transporter transmembrane domains, and reorganized their taxonomy in our classification. A novel case of topological variation among homologs was identified among the LS domains of LeuRS. Finally, we identified a case of distant homology between Caprin-1 and PAN3 dimerization domains. Each of these cases illustrates specifically the more general trend towards large complex structures and the problems of classification of these structures being altogether different from the problems of distinguishing between topologies of soluble globular single-domain proteins.

## Methods

### Classification of the residue covariation-generated model

Coordinates for the CcmB structure model dimer were downloaded from the GREMLIN structure website (https://gremlin2.bakerlab.org/struct.php?page=ccmb). CcmB chain A was queried against the PDB25 representative dataset using the DaliLite server (http://ekhidna2.biocenter.helsinki.fi/dali) [48]. Top hits (Z-score > 7) were mapped to ECOD, and representative structures from hits corresponding to distinct ECOD X-groups (MatE hit: e4mlbB1 [B:3–234] and Type II ABC exporter domain: e5x5yF) were resubmitted as queries against the PDB25 dataset. Select structures from the MatE superfamily (PDB:3mkt and 5 t77) were superimposed with the ABCG5/8 transporter (PDB:5do7), and the conserved core TMH were visualized using PyMol (The PyMOL Molecular Graphics System, Version 2.0 Schrödinger, LLC.).

### Structural searches of type II CAAX proteases

The DaliLite web server was used to identify structurally similar proteins for type II CAAX protease homolog (PDB: 4cad, chain A), APH-1 (PDB: 5a63, chain C), and ABC transporters. For ABC transporters, we used only the transmembrane domains as the query, while other domains such as nucleotide binding domains and extracellular substrate binding domains were excluded. The PDBs, their chains and residue ranges are: FtsX (PDB: 5ws4, chain A, 260–231 and 522–664), ABCG (PDB: 5do7, chain C, 362–649), ABCA (PDB: 5xjy, chain A, 3–46 and 631–846), YjgP_YjgQ (5x5y, chain G, 1–139 and 230–354), BPD_transp_1 (3dwh, chain A, 6–208), and type I exporter (5mkk, chain A, 11–335).

### Generation of LS de novo domain models

For comparison of proteins, three-dimensional structures and their alignments were used DALI [48] and TM-align [49]. For each protein sequence under study a search against the PDB Data Bank [50] and the NCBI non-redundant protein sequence database (National Center for Biotechnology Information, NIH, Bethesda, MD) was performed using BLAST [40] (E-value cutoff < 0.001) and HHpred [51] (E-value cutoff < 0.001). Multiply sequence alignments were performed using MAFFT [52] with BLOSUM62 matrix as the scoring matrix for amino acid sequenes (gap opening penalty = 1.53, offset value = 0.0). De novo structure prediction for leucine-specific domain of *D.phosphitoxidans* LeuRS we did using Rosetta software suite, version 3.9 [41]. For Rosetta simulation fragment files, which contain short backbone fragments that will be randomly inserted at all positions during the simulation, were prepared using Robetta web-server (http://robetta.bakerlab.org). We generated 2000 predicted models which were clustered according to their structure similarity using Calibur [53]. The largest cluster, containing 654 predicted structures, was the focus of study. The most similar structure (RMSD = 2.1 Å) to the *E.coli* LeuRS leucine-specific domain from this cluster was selected to be shown at the Fig. 5f.

## Data Availability

The datasets used and/or analyzed during the current study are available from corresponding author on reasonable request. ECOD domain datasets (current and historical) are available from https://prodata.swmed.edu/ecod.
